# NgcE^Sco^ Acts as a Lower-Affinity Binding Protein of an ABC Transporter for the Uptake of *N,N′*-Diacetylchitobiose in *Streptomyces coelicolor* A3(2)

**DOI:** 10.1264/jsme2.ME17172

**Published:** 2018-09-29

**Authors:** Chiharu Iinuma, Akihiro Saito, Takayuki Ohnuma, Elodie Tenconi, Adeline Rosu, Séverine Colson, Yuuki Mizutani, Feng Liu, Magdalena Świątek-Połatyńska, Gilles P. van Wezel, Sébastien Rigali, Takeshi Fujii, Kiyotaka Miyashita

**Affiliations:** 1 Department of Nanobiology, Graduate School of Advanced Integration Science, Chiba University 648 Matsudo, Matsudo city, Chiba Japan; 2 Department of Materials and Life Science, Shizuoka Institute of Science and Technology 2200–2 Toyosawa, Fukuroi, Shizuoka 437–8555 Japan; 3 Department of Advanced Bioscience, Kinki University 3327–204 Nakamachi, Nara 631–8505 Japan; 4 InBioS—Center for Protein Engineering, Institut de Chimie B6a, University of Liège B-4000 Liège Belgium; 5 Molecular Biotechnology, Institute of Biology, Leiden University Sylviusweg 72, 2333 BE Leiden The Netherlands; 6 National Institute of Agro-Environmental Sciences 3–1–3 Kan-nondai, Tsukuba, Ibaraki Japan

**Keywords:** *N,N′*-diacetylchitobiose, ABC transporter, chitin, chitinase, DasR

## Abstract

In the model species *Streptomyces coelicolor* A3(2), the uptake of chitin-degradation byproducts, mainly *N,N′*- diacetylchitobiose ([GlcNAc]_2_) and *N*-acetylglucosamine (GlcNAc), is performed by the ATP-binding cassette (ABC) transporter DasABC-MsiK and the sugar-phosphotransferase system (PTS), respectively. Studies on the *S. coelicolor* chromosome have suggested the occurrence of additional uptake systems of GlcNAc-related compounds, including the *SCO6005–7* cluster, which is orthologous to the ABC transporter NgcEFG of *S. olivaceoviridis*. However, despite conserved synteny between the clusters in *S. coelicolor* and *S. olivaceoviridis*, homology between them is low, with only 35% of residues being identical between NgcE proteins, suggesting different binding specificities. Isothermal titration calorimetry experiments revealed that recombinant NgcE^Sco^ interacts with GlcNAc and (GlcNAc)_2_, with *K*_d_ values (1.15 and 1.53 μM, respectively) that were higher than those of NgcE of *S. olivaceoviridis* (8.3 and 29 nM, respectively). The disruption of *ngcE^Sco^* delayed (GlcNAc)_2_ consumption, but did not affect GlcNAc consumption ability. The *ngcE^Sco^*-*dasA* double mutation severely decreased the ability to consume (GlcNAc)_2_ and abolished the induction of chitinase production in the presence of (GlcNAc)_2_, but did not affect the GlcNAc consumption rate. The results of these biochemical and reverse genetic analyses indicate that NgcE^Sco^ acts as a (GlcNAc)_2_- binding protein of the ABC transporter NgcEFG^Sco^-MsiK. Transcriptional and biochemical analyses of gene regulation demonstrated that the *ngcE^Sco^* gene was slightly induced by GlcNAc, (GlcNAc)_2_, and chitin, but repressed by DasR. Therefore, a model was proposed for the induction of the chitinolytic system and import of (GlcNAc)_2_, in which (GlcNAc)_2_ generated from chitin by chitinase produced leakily, is mainly transported via NgcEFG-MsiK and induces the expression of chitinase genes and *dasABCD*.

Streptomycetes are multicellular mycelial bacteria that thrive in soil environments as well as in marine and fresh water ecosystems. As producers of a large range of secondary metabolites, including two-thirds of all known antibiotics as well as many anticancer, antifungal and immunosuppressive agents, streptomycetes are of utmost importance for human health, agriculture, and biotechnology (1, 2). Streptomycetes have a saprophytic lifestyle and degrade all naturally occurring biopolymers; therefore, they are a rich source of industrially relevant enzymes (12, 47). These bacteria are major decomposers of chitin, a polymer of beta-1,4-linked *N*-acetylglucosamine (GlcNAc) units. Complete chitin degradation into GlcNAc and *N,N′*-diacetylchitobiose ([GlcNAc]_2_) by streptomycetes requires the production of extracellular chitinases of families 18 and 19 of the glycoside hydrolase (GH) classification (for a review, see [28]), intra- and extracellular *N*-acetyl-β-D-glucosaminidases of GH families 3 and 20 (15, 33, 44), and the lytic polysaccharide monooxygenase of AA10 (21), the amino acid sequence of which is similar to chitin-binding proteins (29, 38).

The uptake of chitin degradation byproducts was initially studied in *Streptomyces olivaceoviridis*, which uses PtsC2, the transmembrane enzyme IIC of the phosphoenolpyruvate phosphotransferase system (PTS), and the ATP-binding cassette (ABC) transporter NgcEFG for GlcNAc uptake (30, 49, 51). NgcEFG also internalizes (GlcNAc)_2_. *S. coelicolor* A3(2) transports GlcNAc via the PTS enzyme IIC NagE2 as a potentially unique uptake system for GlcNAc when this nutrient is provided as the main carbon source (24), while the uptake of (GlcNAc)_2_ is mediated by the ABC transporter DasABC (31) for subsequent hydrolysis into GlcNAc by the *N*-acetyl-β-D-glucosaminidase DasD (33). The catabolism of GlcNAc further requires the GlcNAc kinase NagK, GlcNAc- 6-phosphate deacetylase NagA, and GlcN-6-P deaminase/ isomerase NagB in order to generate fructose-6-phosphate, which will enter glycolysis (39). The expression of all *pts*, *nag*, and *das* genes encoding GlcNAc and (GlcNAc)_2_ transporters and catabolic enzymes is inhibited by the GntR family transcription factor DasR, the DNA-binding activity of which is repressed by GlcNAc-6-P and GlcN-6-P (6, 9, 22, 23, 27, 41, 43). The expression of all of these genes is activated by GlcNAc, except for the *dasA* gene, the transcription of which is induced by chitin and (GlcNAc)_2_ and repressed by GlcNAc (6, 31), similar to the genes encoding chitinase (*chi*) (20). DasR is required for the maximal expression of *chi* genes (22), while in the closely related actinobacterium *Saccharopolyspora erythraea*, DasR acts as a transcriptional repressor of *chi* genes, similar to other chitin/GlcNAc utilization genes (17).

The DasABC system uses the multiple sugar import protein MsiK as an ATPase (32). The inactivation of *msiK* abolishes (GlcNAc)_2_ consumption, whereas the *dasA*-null mutant maintains the ability to consume (GlcNAc)_2_, but at a markedly lower rate (31). These findings suggest that there is at least one additional ABC transporter for the uptake of (GlcNAc)_2_, which also involves MsiK as a common ATPase component (32). In *S. coelicolor*, the MsiK-mediated uptake of (GlcNAc)_2_ is required not only for the utilization of chitin degradation byproducts, but also to induce chitinase production (32). However, the inactivation of *dasA* resulted in stronger total chitinase activity by *S. coelicolor*, which is not consistent with a simple induction model that requires the transport of (GlcNAc)_2_ to trigger the chitinolytic system (6, 31). This phenotype suggests that the proper induction of chitin utilization genes needs to involve diverse sensory/transporter systems that act synergistically or competitively according to the extracellular concentration pattern of chitin-derived nutrients (6).

In order to improve our understanding of the chitin utilization system in streptomycetes, we investigated the role of the *SCO6005–6007* gene cluster of *S. coelicolor*, which has a homologous gene organization and genomic context to the genes for the high-affinity GlcNAc and (GlcNAc)_2_ NgcEFG transporter of *S. olivaceoviridis* (30, 51). However, while gene synteny is conserved, similarities at the amino acid level between *SCO6005–6007* gene products and NgcEFG were low for orthologous proteins. In the present study, we investigated how the lack of similarities between these orthologous transporters impacts on the capacity of the *S. coelicolor* NgcEFG (NgcEFG^Sco^) system to consume and respond to GlcNAc and (GlcNAc)_2_ using biochemical and reverse-genetic analyses.

## Materials and Methods

### Bacterial strains, plasmids, and media

*S. coelicolor* A3(2) strain M145 (14), its *dasA-* and *dasR-*null mutants ASC2 and BAP29 (27, 31), and *dasR*-overexpressing strain carrying the multicopy *dasR* gene (*dasR*^++^) (26) were used. *Escherichia coli* JM109 (52) and DH5α (42) were used as hosts for gene manipulation. *E. coli* ET12567 (*dam dcm hsdS*) (18) was used to prepare plasmids for *S. coelicolor* transformation in order to avoid the methylation-specific restriction system of the bacterium. *E. coli* BL21(DE3)pLysS (Novagen, Burlington, MA, USA) was used to overproduce the NgcE^Sco^ and DasR proteins. The plasmids used in the present study are listed in Table S1. Luria–Bertani (LB) medium (34) was used to culture *S. coelicolor*; *E. coli* transformants were grown in LB medium supplemented with 50 μg mL^−1^ ampicillin or 10 μg mL^−1^ gentamycin. Minimal medium (MM; 10 mM K_2_HPO_4_, 10 mM KH_2_PO_4_, 1 mM CaCl_2_, and 0.5 mM MgCl_2_ supplemented with 0.1% [v/v] trace element solution) (35) was used to investigate the responses of *S. coelicolor* cells to various carbon sources. Soya flour—mannitol (SFM) agar medium (14) was used to prepare spores of *S. coelicolor* strains.

### Gene manipulation

Plasmid preparation and restriction enzyme digestion were performed as described by Sambrook & Russell (2001) (34). DNA fragments were ligated using a DNA ligation kit (Takara Bio, Kusatsu, Japan) according to the manufacturer’s instructions.

### Production and purification of recombinant NgcE^Sco^ and NgcE proteins

Two sets of primers (Table S2) were designed to amplify parts of the *SCO6005* (*ngcE^Sco^*) gene, which encode the part of the NgcE^Sco^ protein without the putative signal peptide (29 amino acids from the N terminus). The recombinant NgcE^Sco^ protein was tagged with an N-terminal 6×His or N-terminal GST using pET16b or pGEX-4T-1 (Table S1). Both recombinant NgcE^Sco^ proteins were successfully overproduced in a soluble form and purified using Ni-NTA agarose (Qiagen, Hilden, Germany) and Glutathione Sepharose 4B (GE Healthcare, Waukesha, WI, USA), respectively. The recombinant N-terminally His-tagged NgcE protein of *S. olivaceoviridis* was also produced in *E. coli* carrying pQEH301 (Table S1) and purified as reported previously (30). The purified His-tagged NgcE^Sco^ protein was used to prepare *anti*-NgcE^Sco^ antiserum, while binding affinities for the sugars of the purified GST-tagged NgcE^Sco^ protein were assessed as described below following the removal of the GST-tag. The sugar-binding affinity of the purified His-tagged NgcE protein was also analyzed as described below. See the Supplementary Materials and Methods for detailed conditions pertaining to protein production and purification.

### Isothermal titration calorimetry (ITC)

ITC experiments were performed with an iTC_200_ system (GE Healthcare) (50). Solutions were thoroughly degassed prior to experiments in order to avoid air bubbles in the calorimeter. A volume of 0.2028 mL of NgcE^Sco^ solution (19 μM) in 20 mM Tris/HCl buffer (pH 8.0) at 30°C was placed in the reaction cell, and ligand solutions in identical buffers were placed in the ITC syringe. In all titrations, 0.8-μL aliquots were injected into the reaction cell at 80-s intervals with a stirring speed of 1,000 rpm. Titrations were completed after 40 injections. The shape of the ITC binding curve was assessed by the Wiseman *c* value. When titration experiments were performed with *c* values from 10 to 100 (*c*=N·*K*_a_·[M]*t*; where N is stoichiometry, *K*_a_ is the association constant, and [M]*t* is the initial protein concentration), the *K*_a_ values obtained were regarded as being reliable (50). ITC data were collected and fit automatically using microcal origin v.7.0 software accompanying the iTC_200_ system (50). All data from the binding reactions fit well with the single-site binding model yielding stoichiometry (N), an equilibrium dissociation constant (*K*_d_), and enthalpy change (Δ*H*). The reaction free energy change (Δ*G*) and entropy change (Δ*S*) were calculated from the relationship described in the following equation: Δ*G*=−*RT*ln*K*_assoc_=Δ*H*−*T*Δ*S*.

### Assessment of binding affinities for sugars based on alterations in fluorescent strength

The *K*_d_ value of NgcE^Sco^ or NgcE was measured against *N*-acetylglucosamine, *N*-acetylgalactosamine, *N*-acetylmuramic acid, glucose, xylose, or mannose based on a fluorescence method (10).

### Disruption of the *ngcE^Sco^* gene

The *ngcE^Sco^* gene was disrupted in the wild-type strain *S. coelicolor* A3(2) M145 and its *dasA*-null mutant ASC2 (31) by homologous recombination using the temperature-sensitive plasmid pAS100 (Table S1) (51). Most of the *ngcE^Sco^* gene was replaced by the *aacC4* gene cassette (Fig. S3 and S4) (3). Detailed methods are described in the Supplementary Materials and Methods.

### Complementation of the *ngcEFG^Sco^* gene cluster

As derivatives of the multi-copy plasmid vector pWHM3 (Table S1) (45), the plasmids pWHM3-*ngcEFG* and pWHM3-*ngcFG* were prepared to express *ngcEFG^Sco^* and *ngcFG^Sco^*, respectively, with the native promoter region (Fig. S3). Details for constructing these plasmids are provided in the Supplementary Materials and Methods. These constructs were introduced into *S. coelicolor* strains via protoplast transformation (14).

### Conditions for the *S. coelicolor* culture

In order to investigate the responses of cells to various sugars, we cultured *S. coelicolor* strains according to a previously described method (31). Spores formed on SFM agar medium were inoculated into 30 mL of LB medium in a 100-mL flask with a spring (14) and grown at 30°C for 18–20 h on a rotary shaker at 150 rpm. Mycelia were harvested by centrifugation (3,000 rpm, 3 min), washed with MM without carbon sources, suspended in 60 mL of MM, and divided into several aliquots. Each aliquot was supplemented with a different carbon source: 250 μM of glucose, maltose, cellobiose, xylobiose, glucosamine, GlcNAc, or (GlcNAc)_2_ and 0.05% (w/v) colloidal chitin. After sugar supplementation, cultures were again grown at 30°C on a rotary shaker at 150 rpm. In measurements of GlcNAc and (GlcNAc)_2_ consumption rates, the amount of mycelia in MM was adjusted to 19–21 mg fresh weight mL culture^−1^. Culture fluids were sampled periodically, centrifuged to separate the supernatant and mycelia, and stored at −80°C. The sugar concentrations and chitinase activities of the supernatants were measured, whereas mycelia were used for total RNA preparation and immunoblot analyses.

### Measurement of sugar concentrations

GlcNAc and (GlcNAc)_2_ concentrations were measured in culture supernatants using high-performance liquid chromatography with UV detection at 215 nm (SPD-20A; Shimadzu, Kyoto, Japan) and a normal phase column of 4.6 mm×250 mm (Inertsil NH_2_ 3 μm; GL Science, Tokyo, Japan). GlcNAc and (GlcNAc)_2_ were separated under isocratic conditions (acetonitrile/water=65/35 [v/v]) at a flow rate of 1.0 mL min^−1^ and identified by their respective retention times.

### Chitinase assay

Chitinase activity was measured using the fluorescent substrate 4-methylumbelliferyl-*N,N′*-diacetylchitobioside (Sigma, St. Louis, MO, USA) according to a previously described method (19). One unit of chitinase activity was defined as the amount of enzyme that liberated 1 μmol of 4-methylumbelliferone from the substrate at 37°C in one minute.

### Electromobility gel shift assays (EMSAs)

EMSAs were performed using Cy5-labeled *dre* probes (final concentration, ~0.1 mM) and DasR-6His (final concentration, ~1 mM) in a total reaction volume of 50 μL. The protocol for DasR-6His production from pFT240 (Table S1) (26) in *E. coli* BL21(DE3) and subsequent purification onto a Ni^2+^-nitrilotriacetic acid-agarose column was applied as previously described (43). Probes were separated by gel electrophoresis in a 1% (w/v) agarose gel and the fluorescence of the probes was visualized using a Typhoon Trio + variable mode imager (GE Healthcare). The sequences of the oligonucleotides used to generate Cy5-fluorescent double-stranded DNA probes (*dre^nagKA^*, *dre^dasA^*, and *dre^nagB^*) are described in Table S2.

### ChIP-on-chip and microarray analysis

ChIP-on-chip and microarray analyses of the DasR binding event on the *ngcE^Sco^* upstream region and the transcription profiles of *ngcE^Sco^*, respectively, were retrieved from raw data published as supplementary files from Świątek-Połatyńska *et al.* (2015) (41).

### Reverse transcription-PCR

DNA-free total RNA was prepared from mycelia using our method (31) and an SV Total RNA Isolation System (Promega, Madison, WI, USA). In order to characterize transcripts, a reverse transcription (RT)-PCR analysis was performed using AccuPower RT/PCR Premix (Bioneer, Daejeon, Korea) as reported previously (31). A set of primers specific for the *ngcE^Sco^* transcript was designed to give a PCR product of 540 bp (Table S2). In PCR, the number of cycles was set to 20 in order to avoid the saturation of PCR product formation. RT-PCR experiments without prior RT were performed in order to ensure that no residual DNA was present in the RNA samples.

In expression studies on *dasA*, *nagE2*, and *ngcE^Sco^* in *S. coelicolor* M145, the RNAs of the *dasR* null mutant (BAP29) and the strain overexpressing *dasR* (*dasR*^++^) were collected after 30 h of growth in MM mannitol (0.5% [w/v]) agar plates with or with 1% GlcNAc. In the semi-quantitative analysis, samples were taken at four-cycle intervals between cycles 27–35 in order to compare non-saturated PCR product formation (amplifications at cycles 27 and 31 are presented in the first and second wells of each assay). RT-PCR without reverse transcription was performed as a control in order to confirm the absence of residual DNA. Data were verified by three independent experiments.

### Immunoblot analysis

*S. coelicolor* mycelia, which were incubated for 4 h in MM supplemented with 250 μM of each carbon, were harvested by centrifugation (18,000×*g*, 4°C, 3 min), suspended in phosphate-buffered saline (34), and disrupted by sonication (15 s×8) on ice. The suspension was centrifuged at 10,000×*g* at 4°C for 5 min, and the protein concentration of the supernatant was measured by Bradford’s method (4). Proteins corresponding to 50 μg were separated with 10% polyacrylamide gels containing 0.1% sodium dodecyl sulfate (16) and blotted onto a polyvinylidene difluoride membrane (Immobilon-P; Millipore, Burlington, MA, USA). *Anti*-DasA antiserum (31) and *anti*-NgcE^Sco^ antiserum, which were prepared using the His-tagged NgcE^Sco^ protein as an antigen, were used in the immunoblot analysis.

## Results

### In silico analysis of *SCO6005–6007* of *S. coelicolor*

*SCO6005* encodes a putative extracellular sugar-binding component of the transporter (pfam01547), the orthologous protein of which in *S. lividans* is exported via the twin-arginine translocation (TAT) pathway (11). The gene cluster includes two additional ORFs encoding the putative ABC-type integral membrane proteins (*SCO6006* and *SCO6007*) that form a transporter permease (Fig. S1). Regarding most streptomycetes sugar ABC transporters, the gene for the ATPase component was not included in the cluster and energy for sugar import was most likely provided by the multiple sugar import ATPase MsiK (13, 32, 36, 37, 46). The Rok family regulatory gene *rok7B7* is immediately downstream of the operon, and controls the xylose operon *SCO6009–6011* (40). Upstream of *SCO6005*, *6004* encodes a putative alpha-1,2-mannosidase.

The *SCO6005–6007* operon of *S. coelicolor* is an orthologue of the *S. olivaceoviridis ngcEFG* operon, which encodes a high-affinity transporter for GlcNAc and (GlcNAc)_2_ (30, 51). While gene synteny is strictly conserved in streptomycetes, identity at the amino acid level between *SCO6005–6007* gene products and NgcEFG is low for orthologous proteins, namely 35% amino acid identity for the SCO6005 protein and NgcE, 44% for SCO6006 and NgcF, and 50% between SCO6007 and NgcG (Fig. S1). In contrast, the other streptomycetes NgcE orthologues share between 80 to 91% amino acid identities throughout the full-length sequence. These low amino acid identities between *S. coelicolor* and *S. olivaceoviridis* and other streptomycetes are limited to the three Ngc proteins because the putative products of adjacent ORFs *SCO6004* and *SCO6008* (ROK7B7) present high levels of identity, as expected for orthologous proteins.

### Binding specificity of the NgcE^Sco^ protein

The lack of identity between Ngc proteins from *S. coelicolor* and *S. olivaceoviridis* prompted us to assess the binding affinity of the solute-binding component of the transporter of *S. coelicolor* (NgcE^Sco^). The binding specificity and affinity of the pure NgcE^Sco^ protein heterologously produced in *E. coli* (see Materials and Methods for details) was initially investigated using ITC. As shown in Fig. S2, the quantity of heat of the NgcE^Sco^ solution increased with the concentrations of GlcNAc and (GlcNAc)_2_, but was not affected by the addition of (GlcNAc)_3_ and higher oligomers up to (GlcNAc)_6_, thereby demonstrating that the recombinant NgcE^Sco^ protein interacted with GlcNAc and (GlcNAc)_2_. NgcE^Sco^ and GlcNAc/(GlcNAc)_2_ bound in a 1:1 stoichiometry and binding in both cases was driven by enthalpy, while the loss of entropy opposed binding, suggesting a specific interaction between NgcE^Sco^ and GlcNAc/ (GlcNAc)_2_ (Table 1). *K*_d_ values for GlcNAc and (GlcNAc)_2_ were 1.15 and 1.53 μM, respectively (Table 1). These values were higher than those of *S. olivaceoviridis* NgcE for GlcNAc and (GlcNAc)_2_ *i.e.*, 8.3 and 29 nM, respectively (51), and that of DasA for (GlcNAc)_2_ *i.e.*, 32 nM (31).

In order to more precisely compare the affinity of NgcE^Sco^ with that of NgcE, the recombinant NgcE^Sco^ and NgcE proteins produced in *E. coli* were purified and their affinities were evaluated based on changes in the fluorescent strengths of the proteins. The addition of GlcNAc did not quench the fluorescent strengths of the proteins, it increased them. *K*_d_ values were calculated based on increments in the fluorescent strength after the addition an increasing amount of GlcNAc. The *K*_d_ value of NgcE^Sco^ for GlcNAc was 1.9 μM, which corresponded with that obtained by ITC (Table 1). The *K*_d_ value of NgcE produced in *E. coli* for GlcNAc was 85 nM. Although this value was one magnitude higher than that obtained by surface plasmon resonance, it was still 22-fold lower than that of NgcE^Sco^, indicating the markedly higher affinity of the NgcE protein. (GlcNAc)_2_ did not modify the fluorescence properties of NgcE^Sco^ or NgcE. The *K*_d_ values of NgcE^Sco^ for *N*-acetylgalactosamine (GalNAc) and *N*-acetylmuramic acid (MurNAc) were 12 and 25 μM, respectively, and were 6- and 13-fold higher than that for GlcNAc (1.9 μM). We also investigated the effects of xylose and mannose on the fluorescent strength of NgcE^Sco^ due to the presence of genes coding for putative mannosidase and a regulator of the xylose operon in the vicinity of the *ngcEFG* operon (Fig. S1). Glucose, xylose, or mannose up to 1 mM did not significantly affect the fluorescent strength of NgcE^Sco^, implying the absence of an interaction between NgcE^Sco^ and these sugars. The *K*_d_ values of the maltose-binding protein (MBP), L-arabinose-binding protein (ABP), and D-glucose/ D-galactose-binding protein (GGBP) of ABC transporters for the corresponding ligand sugars range between 10^−8^ and 10^−6^ M (25). The *K*_d_ values of NgcE^Sco^ for GlcNAc and (GlcNAc)_2_ were in the 10^−6^ M range (Table 1), implying that the protein mediates the uptake of these sugars; however, affinities were lower than those of *S. olivaceoviridis* NgcE for GlcNAc and (GlcNAc)_2_.

### (GlcNAc)_2_ and GlcNAc consumption in the *ngcE^Sco^* mutant

**Fig. 1:**
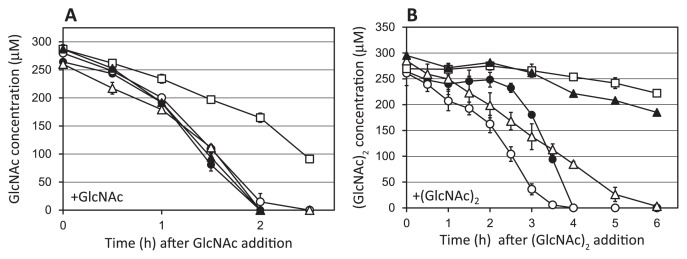
Consumption of GlcNAc and (GlcNAc)_2_ by wild-type and mutant strains of *Streptomyces coelicolor* A3(2). Mycelia grown in LB were washed with minimal medium (MM) without a carbon source, and suspended in MM supplemented with 250 μM GlcNAc (A) or (GlcNAc)_2_ (B). Culture supernatants were sampled periodically and subjected to a HPLC analysis in order to measure GlcNAc and (GlcNAc)_2_ concentrations. Cultivation experiments were performed in duplicate and averages were plotted. Error bars indicate the maximum and minimum values obtained in the duplicate experiment. Open circles, M145; closed circles, CI1 (*ngcE^Sco^*-null mutant of M145); open triangles, ASC2 (*dasA*-null mutant of M145) (31); closed triangles, CI3 (*dasA-ngcE^Sco^* double-null mutant of M145); squares, ASC3 (*msiK*-null mutant of M145) (32). The amounts of mycelia in MM (mg wet weight mL^−1^) were as follows: 21 (M145), 21 (CI1), 19 (ASC2), 20 (CI3), and 21 (ASC3). If necessary, consumption rates were calculated based on the concentrations of the corresponding sugars during the periods in which a linear decline in sugar concentrations was observed.

The *ngcE^Sco^* gene was disrupted in *S. coelicolor* strain M145 and its *dasA* null-mutant ASC2 (Fig. S3 and S4) in order to assess its contribution to GlcNAc and/or (GlcNAc)_2_ uptake. The mycelia of strains M145, ASC2, the *ngcE^Sco^*-null mutant (strain CI1), and *dasA*-*ngcE^Sco^* double-null mutant (strain CI3), pregrown in LB medium, were cultivated in MM supplemented with 250 μM of GlcNAc or (GlcNAc)_2_. GlcNAc consumption rates were not significantly affected by the disruption of *ngcE^Sco^* regardless of whether they were examined in the wild-type- or *dasA*-minus background (Fig. 1A). The disruption of *msiK* lowered the rate of GlcNAc consumption (Fig. 1A), suggesting the presence of ABC transporter(s) for GlcNAc uptake.

In contrast to GlcNAc, the (GlcNAc)_2_ consumption pattern was affected by the *ngcE^Sco^* mutation (Fig. 1B). (GlcNAc)_2_ consumption in the wild-type strain M145 was divided into two steps: the initial step from 0–2 h (2.3 nmol h^−1^ mg mycelia^−1^ [R^2^=0.991]) and the next induced step from 2–3 h (6.0 nmol h^−1^ mg mycelia^−1^ [R^2^=0.998]). In the *ngcE^Sco^*-null mutant, (GlcNAc)_2_ consumption was delayed (Fig. 1B). In the first step during 0–2 h, the (GlcNAc)_2_ concentration remained almost constant, and the initiation of the next induced consumption was delayed for 0.5–1 h. The consumption rate of induced consumption (3–4 h) in CI1 was 8.6 nmol h^−1^ mg mycelia^−1^ (R^2^=0.998). The *dasA*-null mutant ASC2 consumed (GlcNAc)_2_ constantly (2.8 nmol h^−1^ mg mycelia^−1^ [0–5 h, R^2^=0.993]), as reported previously (31).

In the *dasA*-minus background, the effects of the disruption of *ngcE^Sco^* were more obvious. The *dasA*-*ngcE^Sco^* double mutant CI3 showed a low level of (GlcNAc)_2_ consumption (1.2 nmol h^−1^ mg mycelia^−1^ [2–5 h, R^2^=0.986]). The *msiK-*null mutant ASC3 had the lowest consumption rate (0.6 nmol h^−1^ mg mycelia^−1^ [2–7 h, R^2^=0.960]) among the strains tested. These results indicate that the *ngcE^Sco^* gene is involved in (GlcNAc)_2_ uptake in *S. coelicolor* M145, particularly in the initial and constant consumption prior to the induction of the DasABC-MsiK transporter.

### Chitinase production in the *ngcE^Sco^* mutant

**Fig. 2:**
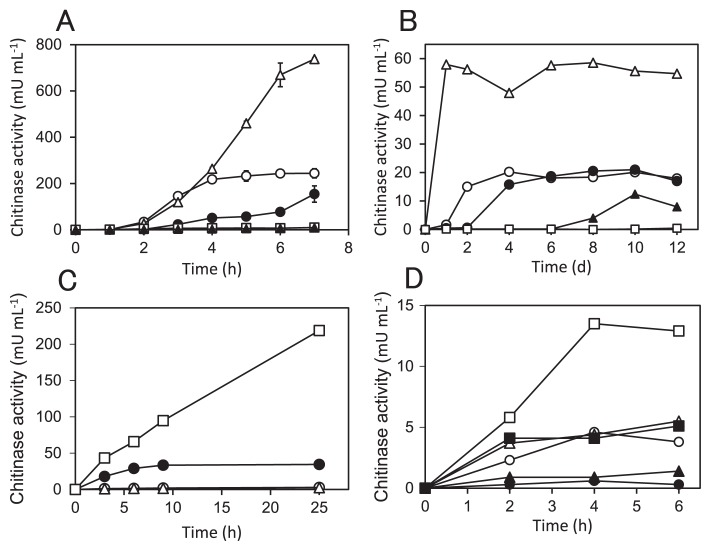
Chitinase activity in the culture supernatant of strains of *Streptomyces coelicolor* A3(2). Mycelia grown in LB were washed with minimal medium (MM) without a carbon source, and suspended in MM supplemented with 250 μM (GlcNAc)_2_ (A and C) or 0.05% (w/w) colloidal chitin (B). In the experiment for C, 5 μg mL^−1^ thiostrepton was added to LB and MM. The culture supernatant was periodically sampled and subjected to a chitinase assay (see Materials and Methods for detailed conditions). (A and B) Open circles, M145; closed circles, CI1 (*ngcE^Sco^*-null mutant of M145); open triangles, ASC2 (*dasA*-null mutant of M145) (31); closed triangles, CI3 (*dasA-ngcE^Sco^* double-null mutant of M145); squares, ASC3 (*msiK*-null mutant of M145) (32). The result shown in A was obtained using the culture supernatants sampled for Fig. 1B. (C) Open circles, CI3 (*dasA-ngcE^Sco^* double-null mutant of M145) carrying the plasmid vector pWHM3; open triangles, CI3 carrying the plasmid pWHM3-*ngcFG* containing *ngcFG^Sco^* with the native promoter; open squares, CI3 carrying the plasmid pWHM3-*ngcEFG* containing the whole *ngcEFG^Sco^* cluster with the native promoter; closed circles, CI1 (*ngcE^Sco^*-null mutant of M145) carrying pWHM3. (D) A total of 5 μM (closed symbols) or 50 μM (open symbols) (GlcNAc)_2_ was added to MM. Circles, M145; triangles, CI1 (*ngcE^Sco^*-null mutant of M145); squares, ASC2 (*dasA*-null mutant of M145) (31).

We previously reported that (GlcNAc)_2_ uptake is necessary for the induction of chitinase production in *S. coelicolor* (32). In order to elucidate the involvement of NgcE^Sco^ in chitinase production, the effects of the disruption of *ngcE^Sco^* on chitinase production were investigated. As shown in Fig. 2A, the chromosomal deletion of *ngcE^Sco^* reduced the level of chitinase activity induced in the presence of (GlcNAc)_2_. In contrast, the *dasA*-*ngcE^Sco^* double mutation fully abolished chitinase production in the presence of (GlcNAc)_2_ (Fig. 2A), as observed for the *msiK*-null mutant ASC3 (32). The *dasA* mutant, which had a lower (GlcNAc)_2_ consumption rate than M145 (Fig. 1A), exhibited stronger chitinase activity (Fig. 2A), as reported previously (31). The delay in chitinase production (Fig. 2B) was reproducibly observed in CI1 when colloidal chitin was added, suggesting that NgcE is involved in sensing chitin and triggering the chitinolytic system. The double mutant CI3 showed partial chitinolytic activity in the presence of colloidal chitin after a prolonged incubation (8–10 d) (Fig. 2B). Complementation experiments revealed that the *dasA-ngcE^Sco^* double mutant CI3 recovered the induction of chitinase production by introducing a multi-copy plasmid carrying *ngcEFG^Sco^* with its native promoter, whereas it did not with a plasmid only carrying *ngcF^Sco^* and *ngcG^Sco^* encoding the membrane component of the transporter (Fig. 2C and S3). Similar to the *dasA* mutant (Fig. 2A), the induced level of chitinase activity was markedly higher in strain CI3, which carries the *ngcEFG^Sco^* operon on a multi-copy plasmid (pWHM3-*ngcEFG*), than in CI1, which is the *ngcE^Sco^* mutant carrying the empty vector (pWHM3) (Fig. 2C). The production of NgcE^Sco^ in complemented strain CI3 (pWHM3-*ngcEFG*) was confirmed by the immunoblot analysis using *anti*-NgcE^Sco^ antiserum (Fig. S5).

In order to elucidate the roles of the distinctive transporters in the induction of the chitinolytic system, we assessed chitinase production profiles in the presence of lower concentrations of (GlcNAc)_2_. At 50 μM of (GlcNAc)_2_, the *dasA* mutant exhibited stronger chitinolytic activities than the parental strain M145 and its *ngcE^Sco^* mutant CI1 (Fig. 2D). At 5 μM of (GlcNAc)_2_, the level of chitinase activity in the *dasA* mutant was similar to that in the presence of 50 μM (GlcNAc)_2_ in M145 and CI3, while the *ngcE^Sco^* mutant and the parental strain M145 exhibited very weak chitinase activities at this concentration (Fig. 2D).

### Regulation of *ngcE^Sco^* expression

**Fig. 3:**
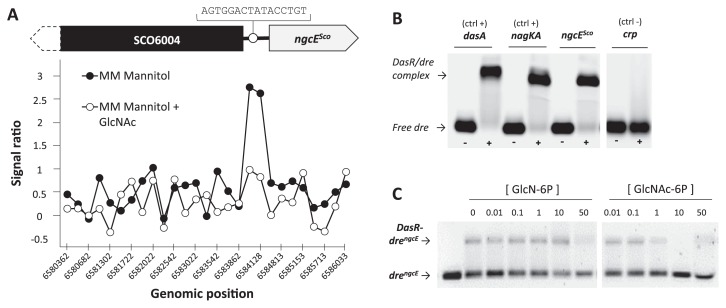
DasR interaction with the *dre* upstream of *ngcE^Sco^*. (A) ChIP-on-chip experiment showing the DasR-binding event upstream of *SCO6005* (*ngcE^Sco^*). Plots show DasR binding in the wild-type grown in minimal medium (MM) mannitol (closed circles) and in MM mannitol with GlcNAc (open circles). Note the absence of a binding event when GlcNAc is added to the medium. Arrows indicate the orientation of the genes adjacent to the DasR-binding signal. (B) EMSA with pure DasR and Cy5 probes centered on *dre* upstream of *ngcE^Sco^*, and *dasA*, and *nagKA* used as positive controls (ctrl +). The probe with the binding site of Crp (7) was used as a negative control (ctrl −). (C) EMSA showing the allosteric effects of both GlcNAc-6P and GlcN-6P on DasR binding to *dre^ngcE^*. The concentrations of both ligands are expressed in mM. In B and C, the final concentration of DasR was set to ~1 mM.

A ChIP-on-chip approach for *S. coelicolor* M145 carrying the integrative vector pGAM29, which expresses C-terminally 3×FLAG-tagged DasR (see [41] for details), revealed DasR binding to the intergenic region between *SCO6004* and *SCO6005* (*ngcE^Sco^*) (Fig. 3A). This region possesses the predicted DasR responsive element (*dre*) AGTGGACTATACCTGT at nt position −334 upstream of *SCO6005* (*dre^ngcE^*) (Fig. 3A), which matches 12 out of the 16 nt of the *dre* consensus sequence (5). The DasR-binding event was abolished when *S. coelicolor* was grown in the presence of GlcNAc (Fig. 3A). In order to confirm ChIP-on-chip data, EMSAs were performed using pure His-tagged DasR (DasR-6His) and a short double-stranded oligonucleotide centered on *dre^ngcE^* (Table S2). DasR interacted with the DNA probe containing *dre^ngcE^*, as observed with the positive control probes containing *dre* upstream of *nagKA* and *dasA* (*dre^nagKA^* and *dre^dasA^*) (Fig. 3B). The binding of DasR to the *dre^ngcE^*-containing probe was inhibited by GlcNAc-6P and GlcN-6P (Fig. 3C). GlcNAc-6P inhibited binding more efficiently than GlcN-6P (Fig. 3C). These results of the ChIP-on-chip analysis and EMSAs were consistent with those reported for the interactions of DasR with other *dre* (41, 43). GlcNAc-derived GlcNAc-6P and GlcN-6P inhibited the binding of DasR to *dre* in the ChIP-on-chip analysis.

**Fig. 4:**
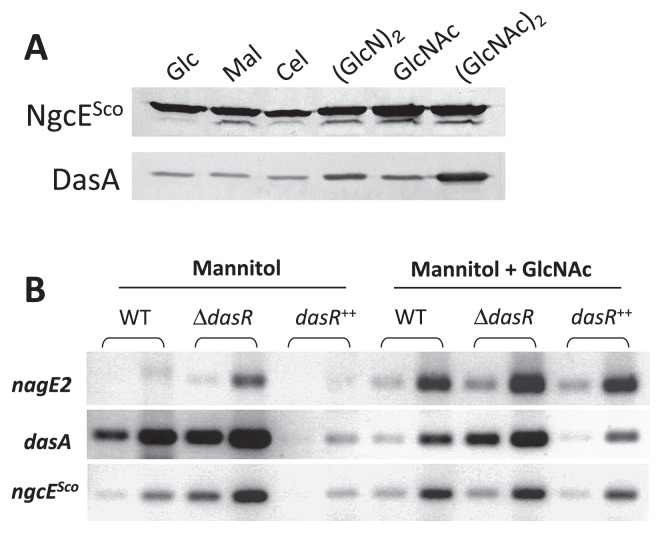
Expression control of *ngcE^Sco^* in *Streptomyces coelicolor* A3(2). (A) The NgcE^Sco^ protein produced in the presence of different mono- and disaccharides. The NgcE^Sco^ protein was detected in the cell lysate of *S. coelicolor* A3(2) mycelia using *anti*-NgcE^Sco^ antiserum. The DasA protein was also detected using *anti*-DasA antiserum for comparisons (31). Abbreviations: Glc, glucose; Mal, maltose; Cel, cellobiose; Xyl, xylobiose; GlcNAc, *N*-acetylglucosamine; (GlcNAc)_2_, *N,N′*-diacetylchitobiose; (GlcN)_2_, chitobiose. (B) Role of DasR and GlcNAc in the expression of *ngcE* analyzed by semi-quantitative RT-PCR. The transcription of the GlcNAc-specific PTS EIIC component *nagE2* (*SCO2907*) or *dasA* (*SCO5232*) was used as a positive control for DasR-dependent and GlcNAc-induced or -repressed genes, respectively. The transcription of 16S rRNA was used as a control for the DasR-independent gene (not shown). RNA samples were collected from *S. coelicolor* M145 (WT, wild-type), the *dasR* mutant BAP29 (Δ*dasR*), and the *dasR* multicopy mutant (*dasR*^++^) grown at 28°C for 30 h (early transition phase) on MM mannitol agar plates with or without 1% GlcNAc. In the semi-quantitative analysis, samples were taken at four-cycle intervals in order to compare non-saturated PCR product formation (amplifications at cycles 27 and 31 are presented in the first and second wells for each assay). Data were verified in three independent experiments. See Table S1 for the oligonucleotides used.

Previous transcriptomic studies also revealed that *ngcE^Sco^* expression was induced by chitin (23) and GlcNAc (41). The microarray analysis revealed that the expression of *ngcE^Sco^* was up-regulated in the *dasR* mutant in the absence of GlcNAc and appeared to be induced at earlier time points (24 and 30 h) when *S. coelicolor* M145 was grown in MM medium supplied with GlcNAc (Fig. S6A). Very similar expression profiles were observed for *ngcF^Sco^* and *ngcG^Sco^* (41), suggesting that *ngcE^Sco^* (*SCO6005*), *ngcF^Sco^* (*SCO6006*), and *ngcG^Sco^* (*SCO6007*) form a tri-cistronic operon that was herein confirmed using the RT-PCR analysis (Fig. S6B). In the *dasR* mutant, *ngcE^Sco^* transcription was not induced by GlcNAc (Fig. S6A). When mycelia grown in LB were exposed to 250 μM glucose, maltose, cellobiose, xylobiose, GlcNAc, or (GlcNAc)_2_, the amounts of *ngcE^Sco^* transcripts were similar among the tested conditions (Fig. S6C), whereas *dasA* transcription was strongly induced in the presence of (GlcNAc)_2_ under the same culture conditions (31, 32). In order to investigate the expression of *ngcE^Sco^* at the level of protein production, an immunoblot analysis was performed using antibodies against the recombinant His-tagged NgcE^Sco^ protein overproduced in *E. coli*. NgcE^Sco^ production was observed in the presence of glucose, maltose, cellobiose, (GlcN)_2_, GlcNAc, or (GlcNAc)_2_ (Fig. 4A). The levels of production in the presence of GlcNAc and (GlcNAc)_2_ were 1.3- and 1.4-fold higher than that in the presence of glucose, respectively. In contrast, DasA production was markedly induced by (GlcNAc)_2_ and by the glucosamine dimer (GlcN)_2_, though to a markedly lower degree (Fig. 4A). Since the abundant carbon and nitrogen sources contained in LB medium may affect *ngcE^Sco^* transcriptional responses to amino sugars, we repeated the expression studies on RNA samples that were prepared from mycelia grown on MM mannitol (0.5% [w/v]) with or without GlcNAc (1.0% [w/v]) at 28°C for 30 h. Under these conditions, the transcription of *ngcE^Sco^* was stronger in the *dasR* mutant and weaker in the *dasR*^++^ strain than in the parental strain M145, demonstrating that DasR acts as a transcriptional repressor of *ngcE^Sco^* under these conditions (Fig. 4B). Similar to that observed for *nagE2*, the transcription of *ngcE^Sco^* was induced when GlcNAc was added to MM mannitol in the wild-type or *dasR*^++^ strain. However, in the *dasR* mutant, *ngcE^Sco^* transcription was not further enhanced in the presence of GlcNAc, as previously observed in a transcriptomic analysis (Fig. S6A) (41).

## Discussion

In the present study, we investigated the role of the *ngcE^Sco^* gene (*SCO6005*) and its encoding protein NgcE^Sco^ in order to assess its contribution to the uptake and catabolism of chitin and its main byproducts GlcNAc and (GlcNAc)_2_. As discussed in the Introduction, we were unable to strictly refer to a previous study performed on *ngcE* in *S. olivaceoviridis* because despite the conserved synteny, the level of identity with NgcE^Sco^ was only 35% (Fig. S1). The lack of amino acid identity between the two orthologues is reflected in the *K*_d_ values of NgcE^Sco^ measured for GlcNAc and (GlcNAc)_2_ (1.15 and 1.53 μM, respectively [Table 1]), which were higher than those of the *S. olivaceoviridis* NgcE protein for GlcNAc and (GlcNAc)_2_ (8.3 and 29 nM, respectively) (51), and the *K*_d_ value of DasA for (GlcNAc)_2_ (32 nM) (31). The expression of *ngcE^Sco^* was constitutive and induced to some extent by GlcNAc and (GlcNAc)_2_, while *dasA* expression was leaky and strongly induced by (GlcNAc)_2_ (Fig. 4B and S6B). The initial (GlcNAc)_2_ consumption rate in M145 (2.3 nmol h^−1^ mg mycelia^−1^) corresponded well with the constant (GlcNAc)_2_ consumption rate (2.8 nmol h^−1^ mg mycelia^−1^) in its *dasA* mutant, whereas the *dasA*-*ngcE^Sco^* and *msiK* mutants had markedly lower rates (1.2 and 0.6 nmol h^−1^ mg mycelia^−1^, respectively) (Fig. 1B). Therefore, we suggest that NgcE^Sco^ acts as the constitutive sugar-binding protein of the ABC transporter NgcEFG^Sco^-MsiK for the uptake of (GlcNAc)_2_ in *S. coelicolor* A3(2), while DasABC-MsiK is the main (GlcNAc)_2_ uptake system, the production of which is strongly induced by (GlcNAc)_2_. When consumption experiments were performed with various amounts of mycelia (5–15 mg mycelia mL^−1^), the effects of the *ngcE^Sco^* mutation on (GlcNAc)_2_ consumption were negligible, in contrast to the disruption of *dasA*, which markedly reduced the (GlcNAc)_2_ consumption rate (data not shown), possibly reflecting the 50-fold higher *K*_d_ value of NgcE^Sco^ for (GlcNAc)_2_ than that of DasA. We assumed that remaining (GlcNAc)_2_ consumption in the *dasA*-*ngcE^Sco^* and *msiK* mutants was due to (GlcNAc)_2_ hydrolysis based on the basal level of extracellular *N*-acetylhexosaminidases and subsequent consumption of GlcNAc.

The reverse-genetic analysis did not indicate the involvement of *ngcE^Sco^* in the uptake of GlcNAc. The NgcEFG^Sco^- MsiK system may not uptake GlcNAc even though NgcE^Sco^ interacts with GlcNAc. The MalE protein, which is the maltose (maltodextrin)-binding protein for the uptake of maltose and maltodextrin in *E. coli*, interacts with ligands and mediates the uptake of sugars. Reduced or oxidized maltodextrins were not transported into cells, but bound to MalE with good affinity (8). Similarly, the “maltodextrin-negative” mutants of MalE only show a marginal decrease in affinity toward maltodextrins, but do not support the transport of maltodextrins in whole cells (48).

In the present study, we observed a reduced GlcNAc consumption rate in ASC3 (Fig. 1A) that lacks the *msiK* gene encoding the common ATPase component for sugar ABC transporters (32). These results imply the presence of ABC transporters for GlcNAc; however, a previous study reported that the NagE2 of PTS may be a unique permease mediating the uptake of GlcNAc in *S. coelicolor* (24).

The presence of higher (DasABC) and lower (NgcEFG^Sco^) affinity uptake systems for (GlcNAc)_2_ in *S. coelicolor* is likely to have a biological meaning. Similarly, in *S. olivaceoviridis*, the uptake of GlcNAc is mediated by two systems, the affinities of which are distinctive: the *K*_m_ value of one system (the PTS system including PtsC2) for ^14^C-labeled GlcNAc is 5 μM, while that of the other system (ABC transporter containing NgcEFG) is 0.48 μM (30, 49).

The *ngcE^Sco^-dasA* double mutation abolished the induction of chitinase production by (GlcNAc)_2_ as the *msiK* mutation (Fig. 2A). These results clearly demonstrated that the uptake of (GlcNAc)_2_ is essential for the induction of chitinase production, as concluded in our previous study (32). It was noteworthy that the single *ngcE^Sco^* and *dasA* mutants exerted contrasting effects on the induction of chitinase production. The disruption of *ngcE^Sco^* reduced the chitinase activity induced by (GlcNAc)_2_, while the *dasA* mutation increased not only the levels of induced chitinase activity in the presence of (GlcNAc)_2_ or colloidal chitin, but also sensitivity to (GlcNAc)_2_ (Fig. 2A, B, and D). This result implies distinct roles for the two (GlcNAc)_2_ transporters. We assume that DasABC acts in the metabolism of (GlcNAc)_2_. The structures of the *ngcEFG^Sco^* and *dasABC* gene clusters imply roles for the encoding ABC transporters for (GlcNAc)_2_ uptake; a gene for the *N*-acetylglucosaminidase DasD hydrolyzing (GlcNAc)_2_ to GlcNAc is present in the *dasABC* gene cluster, whereas such a gene involved in (GlcNAc)_2_ hydrolysis is not clustered with *ngcEFG^Sco^* (Fig. S1). The disruption of *dasD* increased the level of chitinase production in the presence of (GlcNAc)_2_ or chitin (33). The *dasD* mutation may prolong the life of intracellular (GlcNAc)_2_, which induces chitinase production (33). We assumed that the higher sensitivity of the *dasA* mutant to (GlcNAc)_2_ in chitinase production (Fig. 2D) is attributed to the longer life of intracellular (GlcNAc)_2_, which induces the expression of *chi* genes. In contrast, the reduction in chitinase activity induced by (GlcNAc)_2_ in the *ngcE^Sco^* mutant may be ascribed to the shorter life of the disaccharide.

**Fig. 5:**
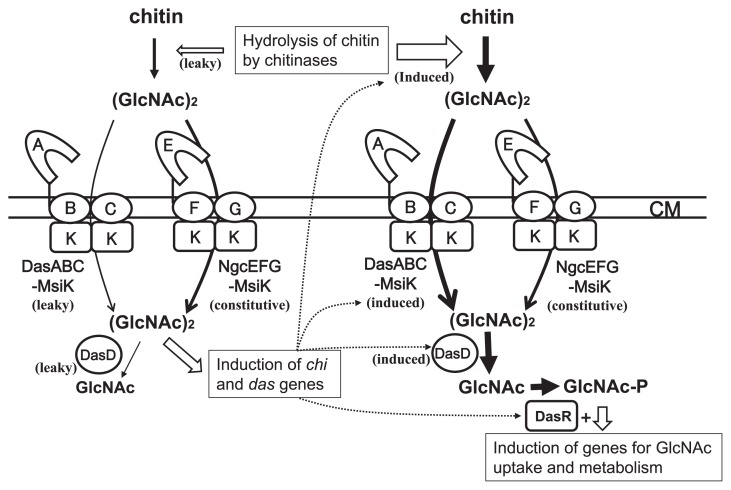
Model of (GlcNAc)_2_ uptake and induction of *das* and *chi* genes in *S. coelicolor* A3(2). (Left part) In the presence of chitin, (GlcNAc)_2_ is continuously generated from chitin by extracellular chitinases produced leakily (or chitinases from other microorganisms in ecosystems), and is promptly taken up via NgcEFG-MsiK and, to a lesser extent, by DasABC-MsiK. This initial uptake unlocks the expression of *das* and *chi* genes. (GlcNAc)_2_ is partially hydrolyzed to GlcNAc by DasD and possibly other intracellular *N*-acetylglucosaminidases (GlcNAcases), which are leakily produced. (Right part) The induced chitinases increase the hydrolysis of chitin in order to generate larger amounts of (GlcNAc)_2_, which is mainly taken up by DasABC, the expression of which is induced by (GlcNAc)_2_. Imported (GlcNAc)_2_ is hydrolyzed to GlcNAc by DasD and other GlcNAcases. GlcNAc is converted to *N*-acetylglucosamine-6-phosphate (GlcNAc-6P) and glucosamine-6-phosphate (GlcN-6P) for its metabolism. GlcNAc-6P and GlcN-6P both interact with DasR in order to release the protein from the *dre* elements, thereby inducing the genes, including those for GlcNAc metabolism.

NgcE^Sco^ did not appear to be essential for the uptake of (GlcNAc)_2_ or induction of chitinase production (Fig. 1B, 2A, and B). However, it is involved in these processes and may have roles in the initial accumulation of intracellular (GlcNAc)_2_ for sensing chitin as a nutrient source in the environment. This hypothesis is supported by the observed late induction of chitinase production in the presence of colloidal chitin and the low initial (GlcNAc)_2_ consumption rate in the *ngcE^Sco^* mutant (Fig. 1B and 2B). In the presence of chitin, (GlcNAc)_2_ is expected to be continuously generated by chitin hydrolysis with extracellular chitinases produced leakily (or possibly by the chitinases of other microorganisms in ecosystems), and continually taken up mainly via NgcEFG^Sco^-MsiK (Fig. 4B) until the (GlcNAc)_2_ concentration becomes sufficient to trigger the expression of *das* and *chi* (Fig. 5). Therefore, the intracellular accumulation of (GlcNAc)_2_ and subsequent induction of chitinase production may be delayed in the *ngcE^Sco^* mutant in the presence of colloidal chitin.

In the *ngcE^Sco^-dasA* mutant, the induction of chitinase production by colloidal chitin was markedly delayed (Fig. 2B). Chitinase production in the presence of colloidal chitin was abolished in the *msiK* mutant (Fig. 2B) (32), which implies the presence of additional ABC transporters for (GlcNAc)_2_ or the heterologous disaccharide GlcNAc-GlcN and/or GlcN-GlcNAc, which may be produced by the hydrolysis of colloidal chitin.

The results of RT-PCR, immunoblot assays, and previous transcriptomic and ChIP-on-chip analyses indicate that the expression of *ngcE^Sco^* is repressed by DasR and induced by GlcNAc, (GlcNAc)_2_, and chitin (22), though with a markedly weaker induction response to these elicitors than *dasA* in the presence of (GlcNAc)_2_ and *nagE2* by GlcNAc (Fig. 3 and 4). It is noteworthy that the control of *ngcE^Sco^* expression is unique because it is the only known DasR-controlled gene that is induced by GlcNAc, (GlcNAc)_2_, and chitin. The *in vivo* binding pattern of DasR to *dre^ngcE^* differed from the patterns of the genes for DasA, chitinases, and GlcNAc metabolism. DasR binding to *dre^ngcE^* was inhibited by the presence of GlcNAc in MM, whereas DasR bound to the *dre* of *dasA* and chitinase genes (*chiA*, *C*, *D*, *H*, *I*, and *J*) (41). In R5 (nutrient rich) medium, DasR binding to the *dre* of the GlcNAc metabolic genes *nagE2* and *nagKA* was inhibited in the presence of GlcNAc, whereas DasR remained bound to *dre^ngcE^* (41). Although we concluded that NgcE^Sco^ acted as a component of the ABC transporter for (GlcNAc)_2_ in the present study, other physiological roles need to be investigated and elucidated.

## SUPPLEMENTARY MATERIALS



## Figures and Tables

**Table 1:** Thermodynamic parameters for *N*-acetylglucosamine (GlcNAc) and *N,N′*-diacetylchitobiose ([GlcNAc]_2_) binding to NgcE^Sco^ obtained from ITC profiles shown in Fig. S2.

Ligand	N	*K*_d_ (μM)	Δ*H* (kcal mol^−1^)	Δ*S* (cal mol^−1^ K^−1^)	−*T*Δ*S* (kcal mol^−1^)	Δ*G* (kcal mol^−1^)
GlcNAc	1.05	1.15	−9.98	−5.77	1.75	−8.23
(GlcNAc)_2_	0.98	1.53	−12.3	−14.0	4.24	−8.06

N, binding stoichiometry; *K*_d_, dissociation constant; Δ*H*, change in enthalpy; Δ*S*, change in entropy; *T*, temperature; Δ*G*, change in Gibbs free energy.
