# Supplementary material for: NgcESco Acts as a Lower-Affinity Binding Protein of an ABC Transporter for the Uptake of N,N′-Diacetylchitobiose in Streptomyces coelicolor A3(2)
Source: Microbes Environ. 2018 Sep 29;33(3):272–81. doi: 10.1264/jsme2.ME17172 (PMC6167110; doi:10.1264/jsme2.ME17172)
Supplement: Supplementary file 1 [file 33_272_s1.pdf]

SUPPLEMENTARY MATERIALS

Supplementary Materials and Methods

Production and purification of the His-tagged and GST-tagged NgcE^{Sco} proteins

The nucleotide sequences for the N-terminal cysteine residue of the presumed mature NgcE^{Sco} protein were substituted with those for a methionine residue in the primer AgcEf (Table S2). Total DNA was prepared from *S. coelicolor* A3(2) M145 by following the method described (6), and the above-mentioned part of the *ngcE*^{Sco} gene was amplified with the primer set by using the total DNA and a KOD plus DNA polymerase (TOYOBO). The PCR product was cloned into the *Sma*I site of the plasmid vector pUC18 (Table S1) to obtain the plasmid pAGC01. The nucleotide sequence of the cloned fragment was ascertained, and the *Nde*I-*Xho*I fragment of pAGC01 was then ligated to the corresponding sites of pET16b (Table S1) to overproduce recombinant DasA protein that was N-terminally tagged with six histidine residues. The resulting plasmid pAGC03 (Table S1) was introduced into *E. coli* BL21(DE3)pLysS (Novagen). The recombinant NgcE^{Sco} protein was successfully overproduced in a soluble form and purified using Ni-NTA agarose (Qiagen) in accordance with manufacturers' instructions. Purified NgcE protein was subjected to *anti*-serum preparation.

To construct a plasimid for producing the C-terminally GST-tagged NgcE^{Sco} protein, a part of *ngcE*^{Sco} was amplified by PCR using a primer set (NgcEpGEX4T1F and NgcEpGEX4T1R. See Table S2) and cloned into the plasmid pGEM-T Easy (Table S1). After asserting the nucleotide sequence of the cloned gene, the *Xho*I-*Eco*RI fragment was integrated into the corresponding sites of the expression plasmid vector pGEX-4T-1 to obtain the plasmid pGEXNgcE (Table S1). *E. coli* BL21(DE3)pLysS carrying the constructed plasmid was cultivated in LB containing 100 μg/ml ampicillin. NgcE^{Sco} production was induced by adding 1 mM IPTG to the culture. *E. coli* cells were harvested by centrifugation and disrupted by sonication on ice. The cytoplasmically soluble fraction, which was obtained by centrifugation, was subjected to Glutathione Sepharose 4B to purify the GST-fused NgcE^{Sco} protein, by following the manufacturer's instructions. To remove the GST-tag, the recombinant NgcE^{Sco} protein was treated with thrombin and subjected to an anion-exchange column (Resource Q, GE Healthcare).

NgcE^{Sco} protein without the GST tag was eluted by an NaCl gradient (0-1 mM) in 20 mM Tris-HCl buffer (pH 8.5).

Disruption of ngcE^{sco} *in M145 and ASC2*

Regions (approximately 1 kb) upstream and downstream of the *ngcE^{Sco}*(*SCO6005*) gene were amplified by PCR using specific primers that we designed (Table S2). The products were cloned into pGEM-T Easy and the sequences of the cloned fragments were confirmed to be identical to those registered in the genome database (http://www.sanger.ac.uk/Projects/S_coelicolor/). The fragment corresponding to the *ngcE^{Sco}* downstream region was isolated with *Hin*dIII and *Kpn*I, and cloned into the corresponding sites of pBlueScript SK+ (Table S1) to obtain the plasmid pDNG03. The *Eco*RI–*Hin*dIII fragment of the *ngcE^{Sco}* upstream region was then inserted into pDNG03 to obtain pDNG04. The *Hin*dIII fragment of the *aacC4* gene cassette (3) was integrated into the corresponding site on pDNG04. A plasmid clone in which the *aacC4* gene was oriented opposite to the residual *ngcE^{Sco}* gene was selected and named pDNG06. pDNG06 was digested with *Eco*RI and *Kpn*I, and the fragment containing the upstream and downstream fragments of *ngcE^{Sco}* and the *aacC4* gene cassette was inserted into the *Eco*RI–*Kpn*I-digested pIJ2925 (5) to obtain pDNG07. The *Bgl*II–*Xba*I fragment of pDNG07, which includes the entire *Eco*RI–*Kpn*I fragment, was inserted into the *Bam*HI–*Xba*I site of the temperature-sensitive vector pAS100 (Table S1) to obtain the *ngcE^{Sco}*-disruption plasmid pDNG08. *S. coelicolor* A3(2) M145 or ASC2 was transformed with pDNG08, which was prepared from *E. coli* ET12567, according to the method described by Kieser *et al*. (2000)(6). After obtaining thiostrepton-resistant transformants at 30°C, we selected strains that grew at 39°C on SFM agar medium supplemented with 10 μg ml^{-1} gentamycin. After streaking the obtained colonies on SFM agar medium containing gentamycin and culturing at 30°C, we obtained strains that were resistant to gentamycin but sensitive to thiostrepton. Disruption of *ngcE^{Sco}* was verified by Southern blot analysis and PCR, using the labeled *ngcE^{Sco}* and *aacC4* genes as probes.

Complementation of the ngcEFG^{Sco} *gene cluster*

The DNA region including the *ngcEFG*^{Sco} gene cluster and its promoter is amplified by PCR using the primer set ngcEFGf and ngcEFGr (Table S2). The PCR product was cloned into the plasmid pGEM-T Easy and its nucleotide sequence was confirmed to be identical to that of the corresponding part of the *S. coelicolor* A3(2) genome (2). The insert obtained by the digestion of the resulting plasmid with *Xba*I and *Hin*dIII was inserted into the corresponding sites of the *E. coli-Streptomyces* shuttle plasmid vector pWHM3 (Table S1) to gain the plasmid pWHM3-*ngcEFG* (Table S1). On the other hand, the *Xba*I-*Hin*dIII fragment containing *ngcEFG*^{Sco} was inserted into the corresponding site of pIJ2925 and the *Cpo*I-*Pst*I region in the *ngcE*^{Sco} of the cloned *ngcEFG*^{Sco} was removed. The both ends of the *Cpo*I-*Pst*I fragment were blunted using a DNA Blunting Kit (Takara Bio) and ligated to obtain the plasmid pIJ2925ΔEFG. The *Xba*I-*Hin*dIII fragment of pIJ2925ΔEFG was cloned into pWHM3 to gain the plasmid pWHM3-*ngcFG* (Table S1). We thus prepared the plasmids pWHM3-*ngcEFG* and pWHM3-*ngcFG* that would express the *ngcEFG*^{Sco} and *ngcFG*^{Sco}, respectively, under the control of the native promoter. The obtained plasmid was introduced to strains of *S. coelicolor* A3(2).

Table S1. Plasmids used in this study.

Name	Description	Reference
pGEM-T Easy	Cloning vector for PCR products in *Escherichia coli*	Promega
pET16b	Expression vector to overproduce N-terminally His-tagged recombinant proteins in *Escherichia coli*	Novagen
pAGC03	A pET16b derivative used for producing N-terminally His-tagged NgcE^{Sco} protein in *E. coli* BL21(DE3)pLysS	This study
pET22b	Expression vector for C-terminally His-tagged recombinant proteins in *Escherichia coli*.	Novagen
pGEX-4T-1	Expression vector to overproduce C-terminally GST-tagged proteins in *E. coli*	GE Healthcare
pGEXNgcE	A pGEX-4T-1 derivative used for producing C-terminally GST-tagged NgcE^{Sco} protein in *E. coli* BL21(DE3)pLysS	This study
pQEH301	A pQE70 (Qiagen, Germany) derivative used for producing N-terminally His-tagged NgcE protein of *Streptomyces olivaceoviridis* in *E. coli*	(9)
pFT240	*dasR* of *S. coelicolor* cloned in pet-22 for producing C-terminally His-tagged DasR in *E. coli* BL21(DE3)pLysS	(8)
pFT241	High-copy shuttle-vector derivative pUWL-SK+ pFT74, in which the *dasR* gene was placed under control of the constitutive glucose kinase gene promoter (P *glkA*)	(8)
pBlueScript SK^{+}	General cloning vector for *E. coli*.	(1)
pUC18	General cloning vector for *E. coli*.	(15)
pIJ2925	pUC18-derived vector having *Bgl*II sites flanking modified multiple cloning sites.	(5)
pAS100	Derivative of the temperature-sensitive plasmid pGM160, from which the *Hin*dIII fragment including the *accC4* gene has been removed. Used for *ngcE* disruption.	(7,14)
pWHM3	*Streptomyces–E. coli* shuttle vector.	(13)
pWHM3-*ngcEFG*	A pWHM3 derivative carrying the *ngcEFG^{Sco}* gene cluster and its native promoter region (Fig. S3).	This study
pWHM3-*ngcFG*	A pWHM3 derivative carrying the *ngcFG^{Sco}* gene cluster and its native promoter region (Fig. S3).	This study

Table S2. Primers used in this study.

Name	Sequence (5′-3′)	Description	Product size (bp)
**For protein production**			
AgcEf	catatgAGCTCTCCCTCCAGCGAG	Used to amplify *ngcE*^{Sco} for production of His-tagged NgcE^{Sco} protein. *Nde*I and *Xho*I sites are underlined in AgcEf and AgcEr, respectively.	1332
AgcEr	ctcgagCTAGCTGACCTTGACCTTG		
NgcEpGEX4T1F	gaattcAGCGACAAGGAGAGCGACAG	Used to amplify *ngcE*^{Sco} for production of GST-tagged NgcE^{Sco} protein. *Eco*RI and *Xho*I sites are underlined in NgcEpGEX4T1F and NgcEpGEX4T1R, respectively.	1323
NgcEpGEX4T1R	ctcgagctaCTAGCTGACCTTGACCTTG		
**For *ngcE*^{Sco} disruption**			
ngcEUf	gaattcTTGTCGACGAGGTTCTCCTTC	Used to amplify the upstream region of *ngcE*^{Sco} for gene disruption. *Eco*RI and *Hin*dIII sites are attached (underlined) in ngcEUf and ngcEUr, respectively.	917
ngcEUr	aagcttACGAATGGTCATCTTTGCATC		
ngcEDf	aagcttGACAAGGTCAAGGTCAGCTAG	Used to amplify the downstream region of *ngcE*^{Sco} for gene disruption. *Hin*dIII and *Kpn*I sites are attached (underlined) in ngcEDf and ngcEDr, respectively.	1057
ngcEDr	ggtaccAGGTGCTTTCCTCTTGCTCTC		
**For reverse-transcription PCR**			
RTAGCf	GCAAAGATGACCATTCGTGCC	Used to detect the *ngcE*^{Sco} gene and its transcripts.	540
RTAGCr	CACCGTCATCACGTAGTTGAG		
msiKf	ATGGCCACTGTTACGTTCGACAAG	Used to detect the *msiK* gene (10).	483
msiKRTr	CTCGTCCATGAGGAACACCTG		
dasAf	CCGAGTCCCTCAACAAGTCC	Used to detect mRNA of *dasA*	200
dasAr	GGGCAGGTAGATCGGCTCGGCG		
nagE2f	GGCTGGGACAAGGTCGCCGCGGTC	Used to detect mRNA of *nagE2*	260
nagE2r	CCCATGATGATGCCGCCGAGCACC		
ngcEf1	AGGAGGCGAAGAAGAAGGGCAAG	Used to detect mRNA of *ngcE*^{Sco}	389
ngcEr1	ACCGGGGACGAACATCTTCTGCTTG		
ngcEf2	ACGGGCTGAACACCGACCAGC	Used to detect mRNA overlapping	246

ngcFr1	GTCGAACGTGAGCTTGCGGCG	*ngcE*^{Sco} and *ngcF*^{Sco}	
ngcFf1	CCTCGTCAACCGCCTCACGGG	Used to detect mRNA overlapping *ngcE*^{Sco} and *ngcF*^{Sco}	296
ngcGr1	AAGGATCTCGCCGGTCGATTT		
**For Electromobility gel shift assays (EMSAs)***			
dre^{nagKA}	CGTACACCCGGGA*GAGGTCTAGTCCACT*GCGGTGGTGTAG	*dre*^{nagKA} fluorescent probe for EMSA (11)	40
dre^{dasA}	CAAGCTCCCCGT*ACTGGTCTACACCATT*GGTCCAGGTCCC	*dre*^{dasA} fluorescent probe for EMSA (11)	40
dre^{ngcE}	CCGCCAAGCGAA*AGTGGACTATACCTGT*CGGCACTCCGGG	*dre*^{ngcE} fluorescent probe for EMSA	40
crp	TGCGGCATCCT*TGTGACAGATCACA*CTGTTTGGACT	Crp-binding site as negative control for EMSA (4)	36
**For amplification of the *ngcEFG*^{Sco} genes**			
ngcEFGf	tctagaACAACGTTGTCAAATCAACTG	Used to amplify the *ngcEFG*^{Sco} cluster and its upstream region including its putative promoter	4063
ngcEFGr	aagcttGCCCTTGATAGAAATCGGTTC		

Sequences corresponding to native sequences are shown in uppercase, whereas introduced nucleotides are indicated in lowercase. In oligonucleotides for EMSAs, sequences bound by the transcription factors are italicized. *Oligonucleotides used for EMSA are labeled with Cy5. Only one oligonucleotide per probe is presented, the complementary oligonucleotide (without Cy5 labelling) is used for generating double stranded DNA probes.

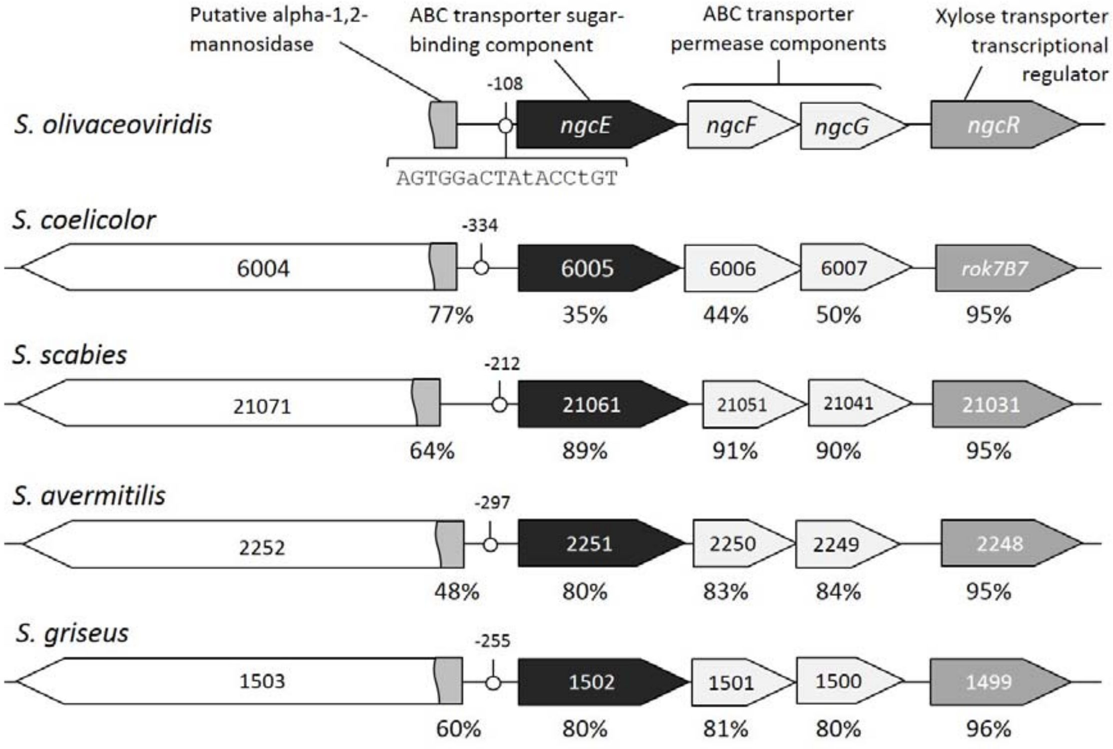

Fig. S1. Synteny of the *ngcEFG* operon in model *Streptomyces* species. The percentages of identity with the corresponding proteins of *S. olivaceoviridis* are displayed below ORFs. Note that only the beginning of the ORF upstream of NgcE has been cloned in *S. olivaceoviridis.* The open circles indicate the position of the DasR-binding sites identified using PREDetector software (12). Numbers inside arrows representing ORFs stand for Gene IDs in *S. coelicolor*, *S. scabies*, *S. avermitilis*, and *S. griseus*.

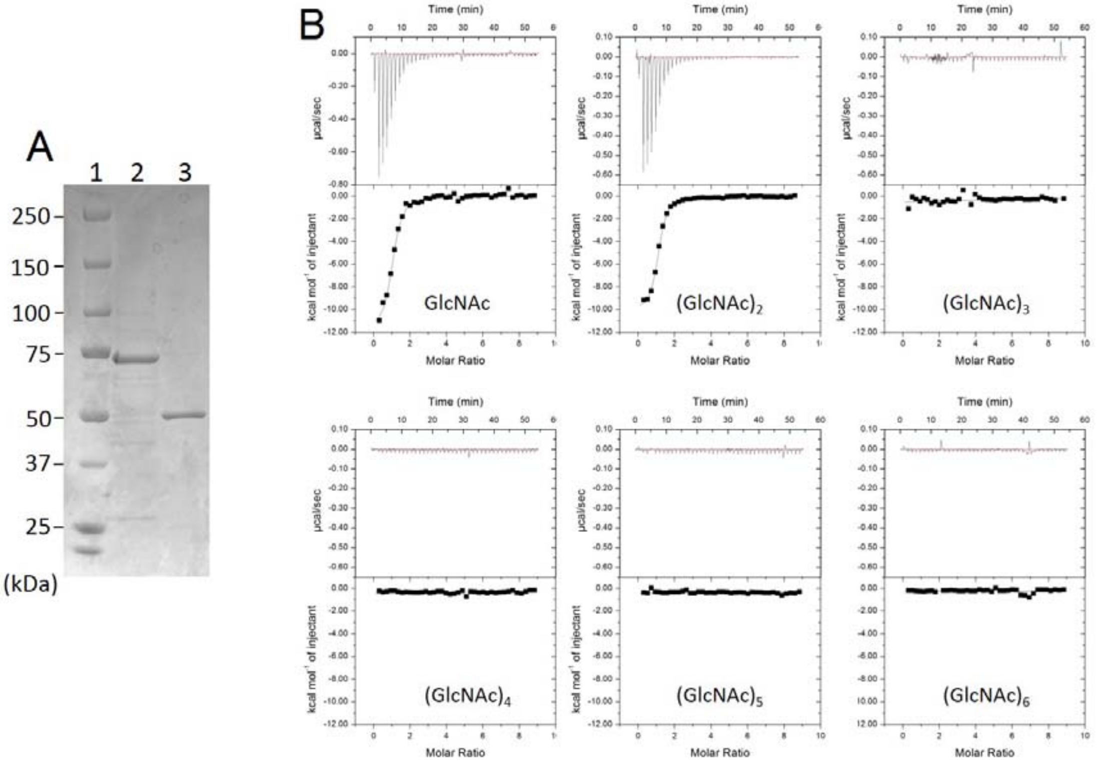

Fig. S2. Isothermal titration calorimetric (ITC) analysis of the recombinant $NgcE^{Sco}$ protein. (A) Purified $NgcE^{Sco}$ protein electrophoresed on an SDS-polyacrylamide gel. Proteins were stained with Coomassie Brilliant Blue. Lane 1, size marker proteins; Lane 2, GST-tagged $NgcE^{Sco}$ protein partially purified using glutathione sepharose resin; lane 3, purified recombinant $NgcE^{Sco}$ protein used for ITC analysis, which was obtained by cleaving the GST-tagged $NgcE^{Sco}$ with thrombin. Approximate molecular masses (kDa) of size marker proteins are also indicated. (B) ITC thermograms and theoretical fits for the interaction of the recombinant $NgcE^{Sco}$ protein with *N*-acetylglucosamine (GlcNAc) and its di- and oligomers $(GlcNAc)_2$, $(GlcNAc)_3$, $(GlcNAc)_4$, $(GlcNAc)_5$, and $(GlcNAc)_6$.

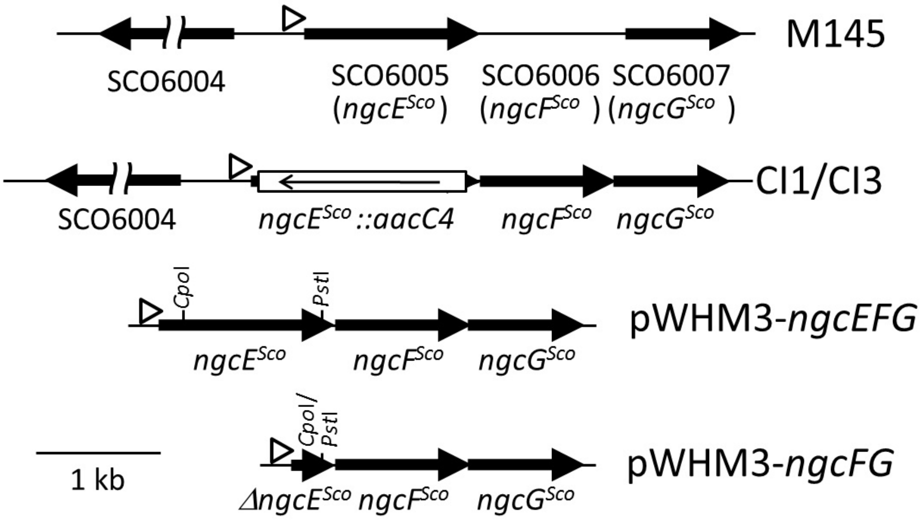

Fig. S3. The map of the *ngcEFG*^{Sco} gene cluster on the *S. coelicolor* A3(2) M145 genome (top) compared with those of the *ngcE*^{Sco} mutant CI1 and the *ngcE*^{Sco}*-dasA* double mutant CI3, and the plasmids pWHM3-*ngcEFG* and pWHM3-*ngcFG*. The position of the native promoter upstream of *ngcE*^{Sco} is indicated by white triangles. A scale bar (1 kb) is shown. The map of M145 was drawn from the data available on the *S. coelicolor* annotation server (http://streptomyces.org.uk/cgi-bin/sco/dc2.pl?width=900&start=4313753&end=4353753).

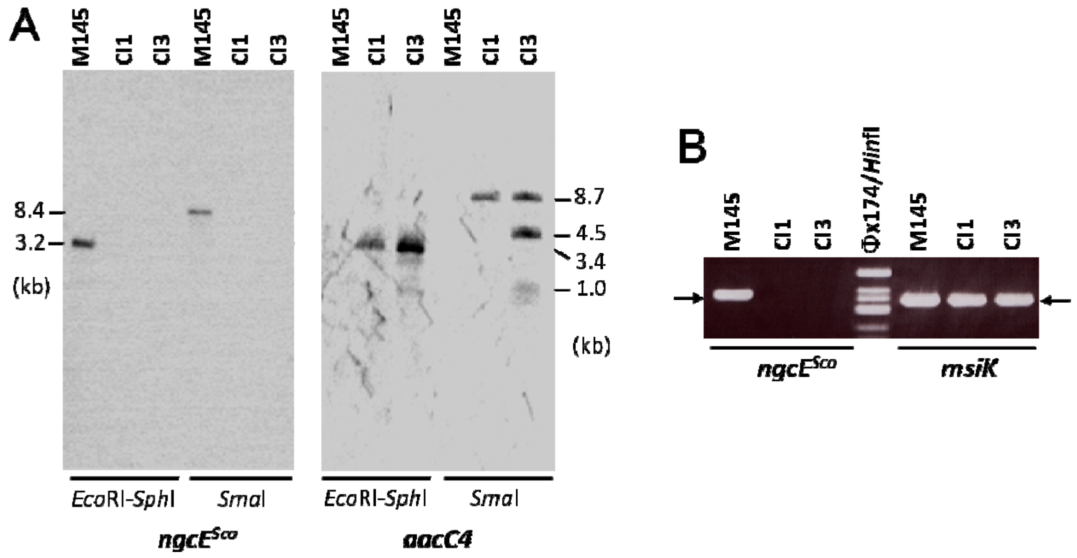

Fig. S4. (A) Sourthern-blot analysis of the total DNA of *S. coelicolor* A3(2) M145, and its *ngcE*^{Sco}-null mutant CI1 and *ngcE*^{Sco}*-dasA* double null mutant CI3. Total DNAs were digested with *Eco*RI-*Sph*I or *Sma*I. The *ngcE*^{Sco} (left) and *aacC4* (right) genes were used as probes. Approximate sizes of the main detected bands are indicated. (B) PCR analysis of the total DNA of *S. coelicolor* A3(2) M145, and its *ngcE*^{Sco}-null mutant CI1 and *ngcE*^{Sco}*-dasA* double null mutant CI3. The primer sets for the amplification of parts of *ngcE*^{Sco} and *msiK* (Table S2) were used for PCR. Positions of the expected sizes of the PCR products are indicated by arrows.

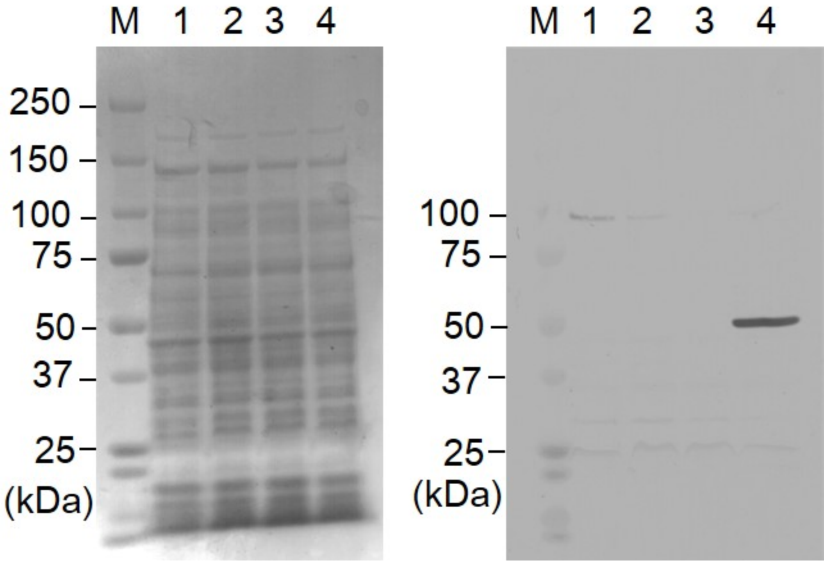

Fig. S5. The NgcE^{Sco} protein production in the presence of $(GlcNAc)_2$. The NgcE^{Sco} protein was detected in the cell-lysate of mycelia of *S. coelicolor* A3(2) transformants by using *anti*-NgcE^{Sco} antiserum. Cell lysates containing 50 μg proteins were electrophoresed on an SDS-polyacrylamide gel in duplicate, and a half of the gel containing one of the duplicate was stained with Coomassie Brilliant Blue (A) and the remaining half was subjected to immuno-blot analysis with the *anti*-NgcE^{Sco} antiserum (B). M, protein size markers; lane 1, CI1 (*ngcE*^{Sco}-null mutant of M145) carrying the plasmid vector pWHM3; lane 2, CI3 (*dasA*-*ngcE*^{Sco} double null mutant of M145) carrying pWHM3; lane 3, CI3 carrying the plasmid pWHM3-*ngcFG* which contains *ngcFG*^{Sco} with the native promoter; lane 4, CI3 carrying the plasmid pWHM3-*ngcEFG* which contains the whole *ngcEFG*^{Sco} cluster with the native promoter. The approximate molecular masses (kDa) of the protein size markers are indicated.

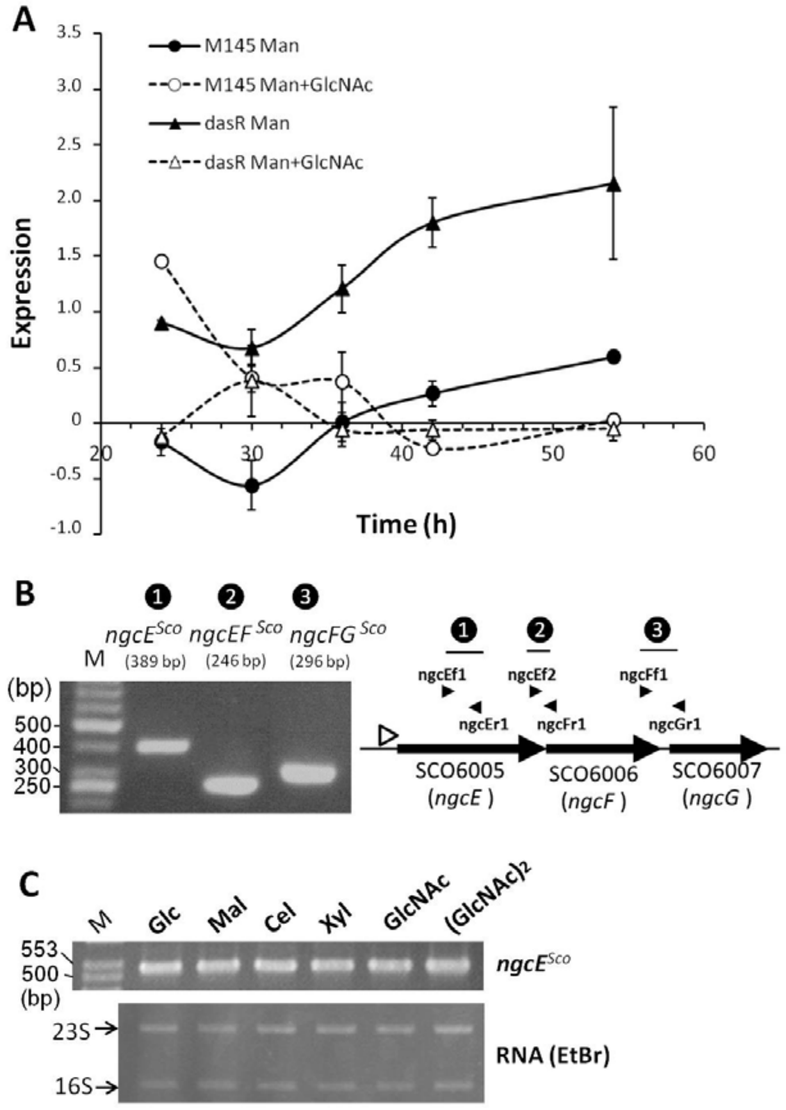

Fig. S6. (A) Expression of *ngcE*^{Sco} deduced from microarray experiments using RNA extracted from the *dasR* mutant GAM29 and its parent *S. coelicolor* M145 grown in MM mannitol with or without a supply of GlcNAc. Time (h) indicates time after inoculation. (B) Detection of transcripts of the *ngcEFG*^{Sco} gene cluster by reverse transcription (RT) PCR. Length of size markers are indicated (bp). The names of primers and the expected sizes of PCR products are also shown. The approximate positions of primers in the *ngcEFG*^{Sco} gene cluster are indicated in the gene map. (C) The *ngcE*^{Sco} transcripts detected by RT-PCR (upper) according to the carbon source supplied, and RNAs used as templates stained with ethidium bromide (EtBr) (lower). Lengths of DNA size markers are indicated (bp). See Table S2 for primer information.

References

1. Alting-Mees, M.A., and J.M. Short. 1989. pBluescript II: gene mapping vectors. Nucleic Acids Res. 17:9494.

2. Bentley, S.D., K.F. Chater, A.M. Cerdeno-Tarraga , *et al*. 2002. Complete genome sequence of the model actinomycete *Streptomyces coelicolor* A3(2). Nature 417:141–147.

3. Blondelet-Rouault, M.H., J. Weiser, A. Lebrihi, P. Branny, and J.L. Pernodet. 1997. Antibiotic resistance gene cassettes derived from the omega interposon for use in *E. coli* and *Streptomyces*. Gene 190:315-317.

4. Derouaux, A., D. Dehareng, E. Lecocq, *et al*. 2014. Crp of *Streptomyces coelicolor* is the third transcription factor for the large CRP-FNR superfamily able to bind cAMP. Biochem. Biophys. Res. Commun. 325:983-990.

5. Janssen, G.R., and M.J. Bibb. 1993. Derivatives of pUC18 that have *Bgl*II sites flanking a modified multiple cloning site and that retain the ability to identify recombinant clones by visual screening of *Escherichia coli* colonies. Gene 124:133-134.

6. Kieser, T., M.J. Bibb, M.J. Buttner, K.F. Chater, and D.A. Hopwood. 2000. *Practical Streptomyces genetics*. Norwich: John Innes Foundation.

7. Muth, G., B. Nußbaumer, W. Wohlleben, and A. Pühler. 1989. A vector system with temperature-sensitive replication for gene disruption and mutational cloning in streptomycetes. Mol. Gen. Genet. 219:341-8.

8. Rigali, S., M. Schlicht, P. Hoskisson, H. Nothaft, M. Merzbacher, B. Joris, and F. Titgemeyer. 2004. Extending the classification of bacterial transcription factors beyond the helix-turn-helix motif as an alternative approach to discover new *cis/trans* relationships. Nucleic Acids Res. 32:3418-26.

9. Saito, A., and H. Schrempf. 2004. Mutational analysis of the binding affinity and transport activity for *N*-acetylglucosamine mediated by the novel ABC transporter Ngc within the chitin-degrader *Streptomyces olivaceoviridis*. Mol. Genet. Genomics 271:545-553.

10. Saito, A., T. Fujii, T. Shinya, N. Shibuya, A. Ando, and K. Miyashita. 2008. The *msiK* gene, encoding the ATP-hydrolyzing component of *N,N'*-diacetylchitobiose ABC transporters, is essential for induction of chitinase production in *Streptomyces coelicolor* A3(2).

Microbiology 154:3358-65.

11. Świątek, M.A., E. Tenconi, S. Rigali, and G.P. van Wezel. 2012. Functional analysis of the *N*-acetylglucosamine metabolic genes of *Streptomyces coelicolor* and role in control of development and antibiotic production. J. Bacteriol. 194:1136-44.

12. Tocquin, P., A. Naome, S. Jourdan, S. Anderssen, S. Hiard, G.P. van Wezel, M. Hanikenne, D. Baurain, and S. Rigali. 2017. PREDetector 2.0: Online and enhanced version of the prokaryotic regulatory elements detector tool. doi: https://doi.org/10.1101/084780.

13. Vara, J., M. Lewandowska-Skarbek, Y.G. Wang, S. Donadio, and C.R. Hutchinson. 1989. Cloning of genes governing the deoxysugar portion of the erythromycin biosynthesis pathway in *Saccharopolyspora erythraea* (*Streptomyces erythreus*). J. Bacteriol. 171:5872-5881.

14. Xiao, X., F. Wang, A. Saito, J. Majaka, A. Schlösser, and H. Schrempf. 2002. The novel *Streptomyces olivaceoviridis* ABC transporter Ngc mediates uptake of *N*-acetylglucosamine and *N,N'*-diacetylchitobiose. Mol. Genet. Genomics 267:429-439.

15. Yanisch-Perron, C., J. Vieira, and J. Messing. 1985. Improved M13 phage cloning vectors and host strains: nucleotide sequences of the M13mp18 and pUC19 vectors. Gene 33:103-119.
